# Entropy-Based Method to Evaluate Contact-Pressure Distribution for Assembly-Accuracy Stability Prediction

**DOI:** 10.3390/e21030322

**Published:** 2019-03-25

**Authors:** Xiao Chen, Xin Jin, Ke Shang, Zhijing Zhang

**Affiliations:** School of Mechanical Engineering, Beijing Institute of Technology, Beijing 100081, China

**Keywords:** contact pressure, entropy, assembly accuracy, assembly-accuracy stability

## Abstract

Assembly accuracy and accuracy stability prediction are significant research directions for improving the reliability and efficiency of precision assembly. In this study, an improved method for assembly accuracy stability prediction, based on the contact-pressure distribution entropy, is presented. By using the contact-pressure distribution as the evaluation parameter instead of the strain-energy distribution, the improved method can not only predict the assembly accuracy of precision assembly more efficiently, but also predict the stability of the assembly accuracy with variations in the ambient temperature. The contact pressure has a clearer mechanical significance than strain energy density in the assembly process, which can be used to distinguish the actual contact area from the contact surface. Hence, the improved method is more efficient and accurate than the original. This study utilizes the same case used in the original method and an additional case from the actual production process to verify the improved method. The correctness and validity of the improved method are proved by these case studies.

## 1. Introduction

Precision assembly is an important research direction in the field of precision mechanical systems, which is receiving increasing attention from researchers. Generally, precision assembly studies mainly focus on precision instruments, precision machine tools, high-precision robots, precision bearings, etc. However, as the assembly accuracy significantly affects the performance of precision equipment, it is crucial to evaluate the assembly quality. For mostly contact-based assemblies, the assembly accuracy can be described as the relative positional relationship between two assembled parts. As the two assembled parts are further deformed due to changes in the ambient temperature or in the external force, the assembly accuracy of the parts will also change accordingly, that is, the assembly accuracy is unstable. Assembly error is the most direct reflection of the assembly accuracy, and is therefore extensively used for evaluating it. Assembly-error variation can directly alter the assembly accuracy stability. Therefore, finding a suitable method to quantitatively evaluate assembly-error variation can significantly improve the reliability of precision assembly.

Existing assembly error evaluation methods mainly include the tolerance analysis method [1,2,3,4,5] and finite element analysis [6,7,8,9,10]. In precision assembly, surface topography errors in the parts are unavoidable and cause assembly error. Of late, research on the abovementioned methods has focused on the effect of surface topography errors on the assembly process [3,4,5,8,9,10]. The surface topography error of the part can be mainly divided into three types: form error, waviness, and surface roughness. Waviness and surface roughness are generally on a micron scale, with limited impact on millimeter-scale parts. Therefore, the effect of waviness and surface roughness on the assembly accuracy is generally considered for assembly processes below the millimeter scale. The scale of the form error is greater than those of the waviness and surface roughness; therefore, studies are increasingly considering the form error in assembly-error analysis. Some researchers have introduced a randomly generated form error surface into traditional 3D tolerance analysis, called the skin model [3,4,5], and have utilized this model to obtain tolerance analysis results closer to the actual assembly situation. Even in the finite element analysis of the assembly process, some researchers have used a 3D model with a randomly generated form error surface [8,9,10] to obtain assembly-error results closer to the actual assembly process, compared to an ideal surface model.

The aforementioned studies have assumed that the form error surfaces are randomly generated, and vast amounts of surface data were used to obtain conclusions for application to the product design process. The tolerance design of products can be planned to an extreme. However, in the processed parts, the surface topography does not generally change during the assembly process. Therefore, it is no longer appropriate to use fitting data to analyze assembly errors in the assembly process. Recently, several researchers have measured the form error on assembly parts and included them in the analysis of assembly errors [11,12,13,14]. Among them, Fang et al. [11] proposed an assembly accuracy prediction method using the actual measured surface. This method uses the strain energy distribution entropy of the contact surfaces, which are form error surfaces obtained from the measurement of the actual parts, to predict the assembly accuracy. However, there are certain problems in the follow-up research and application of this method—three-level evaluation is used in the method, and the process is cumbersome. Further, the final evaluation index is obtained from the comprehensive index of the three-level strain energy distribution entropy. This comprehensive index is derived by comprehensively considering the artificial experience and material parameters, which brings human factors into the prediction process. For contact surfaces with form errors, the form error distribution is the fundamental reason that the error model is closer to the actual assembly process, compared to the traditional ideal surface model. However, this method uses the strain energy distribution as the evaluation parameter; the strain energy only represents the distribution of the contact deformation, and cannot represent the distribution of the actual contact area. This renders the evaluation effect of this method in the actual process, unsatisfactory. The three-level method is selected to obtain the final prediction results.

Contact pressure is a parameter in contact mechanics and has a wide range of engineering applications. Contact pressure distribution can affect the friction properties of the contact interface [15], so it is also used in the design of the bearing [16,17]. Zhang et al. [18] designed a safety factor based on the contact pressure for the interference assembly of the gears, which improves the assembly accuracy of the gear assembly. Peng et al. [19] conducted a finite element simulation study on the assembly of fuel cells. The assembly error affects the assembly performance of the fuel cells by affecting the distribution of contact pressure. The study also gives the maximum assembly error allowed for fuel cell assembly. Deguchi et al. [20] developed a pressure sensitive sensor to measure the pressure distribution in imprint lithography. The pressure distribution on the wafer surface can affect the deformation of the wafer and cause dimensional errors on the pressed wafer. In the bolt connection, the connection strength and deformation of connected parts are affected by the contact pressure distribution [21,22,23]. Contact pressure distribution affects the thermal conductivity of the contact surface and is also used in thermal error analysis of machine tool assemblies [24,25]. Tyfour et al. [26] conducted an experimental study on the contact fatigue of the rail and found that the maximum contact pressure is the main reason for the failure of the rail contact surface. In the above studies, the contact pressure has two main effects on the contact surface. On one hand, it affects the contact deformation so that the relative positional relationship between the contact surfaces changes, such as [15,16,17,18,19,20]. On the other hand, it affects the mechanical strength of the connected parts by affecting the transmission of the contact force between the contact faces, thus affecting the ability of the assembled part to resist deformation caused by external forces, such as [21,22,23,24,25,26].

In summary, the aforementioned studies focus only on the assembly accuracy of the assembly process, and not on the assembly-error changes caused by the combined effects of ambient temperature and uneven contact force. In this study, based on the method presented in [11], a more efficient and concise prediction method for the assembly-accuracy stability is proposed. By using the contact-pressure distribution entropy as the evaluation parameter instead of the strain energy distribution entropy, the contact relationship between the evaluation parameter and nonideal surface is reflected directly. Compared to the original method, the improved method has better assembly-accuracy prediction efficiency, and can also predict the stability of the assembly accuracy, in addition to the assembly accuracy. The remainder of this paper is structured as follows: Section 2 presents the improved prediction method; Section 3 compares the prediction results with that of the original method using the same example, and an example from the actual production process is used to further verify the method; and Section 4 compares the difference between the contact pressure and strain energy distributions on nonideal surface contacts. Finally, the proposed method is summarized in Section 5.

## 2. Methods

### 2.1. Contact Pressure

Contact pressure is a critical parameter in contact mechanics. As per the theory of contact mechanics, the contact pressure is the component of the contact force between two contact surfaces along the normal direction of the surface [27]. The normal contact force can be calculated as follows:

(1) $$P=\int_{S}pdS$$

where *P* is the normal contact force, *p* is the contact pressure, and *S* is the local contact area.

If the contact pressure between the contact surfaces is obtained, the contact area can be calculated. As per the Hertzian contact theory, the contact pressure of two hemispheres is proportional to the contact area:

(2) $$p_{m}\propto \frac{a\left(1/R_{1}+1/R_{2}\right)}{E_{1}/E_{2}}$$

where *p_m_* is the average contact pressure, *a* is the linear size of the contact area, *R_1_* and *R_2_* are the radii of the hemispheres, and *E*_1_ and *E_2_* are the Young’s moduli of the hemispheres, respectively.

It can be seen from the above formula that when the contact area is zero (the two surfaces are not in contact), the contact pressure is zero. Therefore, the contact pressure can be used to judge the actual contact state between the contact surfaces. Although the contact pressure is a vector, its direction always follows the normal direction of the local surface. Therefore, it can be used as a scalar without considering the direction.

### 2.2. Contact Pressure Evaluation Method 

The maximum entropy principle is extensively applied in many disciplines [28,29,30,31,32]. In information theory, if there is a discrete random variable, *X*, with possible values of {*x1, x2, …, xn*}, and probability mass function, *P(X*), the entropy, *H,* of *X* is defined as follows:

(3) $$H\left(X\right)=E[\log_{r}(\frac{1}{x_{i}})]=-\sum_{i=1}^{n}p_{i}\log_{r}(p_{i})$$

where *E* is the expectation operator, *r* is the logarithmic base, which generally takes a value of two [33,34,35] (in this study, *r* = 2). When the probability of each random variable, *x_i_*_,_ is the same, i.e., *p_1_ = p_2_=... = p_n_ =* $\frac{1}{n}$, the maximum value of the entropy function, *H _(X)_,* is obtained, and the corresponding maximum value can be calculated as

(4) $$H_{max}=-\sum_{i=1}^{n}\frac{1}{n}\log_{2}\frac{1}{n}=\log_{2}n$$

From the condition of maximum entropy, when the random variable probabilities are identical, the maximum value of the entropy function is obtained. A more disorderly system indicates that the probabilities of all values of the random variable are closer to each other, thereby resulting in higher entropy. Therefore, entropy can be used to evaluate probability uniformity of the values of a random variable.

As previously mentioned, the contact pressure can be treated as a scalar. If there are a series of nodes on the contact surface and the contact pressure value of node, *i*, is *c_i_*, the contact pressure can be normalized as

(5) $$C\left(x\right)=\frac{c_{i}}{\sum_{i=1}^{n}c_{i}}$$

Based on this, the contact pressure entropy, *H_c_*, can be expressed as

(6) $$H_{c}=-\sum_{i=1}^{n}C_{i}\log_{2}C_{i}$$

The corresponding maximum entropy, *H_max_*, can be expressed as

(7) $$H_{max}=\log_{2}n$$

The normalized entropy, *H_cn_,* can be expressed as

(8) $$H_{cn}=\frac{H_{c}}{H_{max}}$$

The greater the normalized contact pressure entropy, *H_cn_*, the more uniform is the contact pressure distribution.

### 2.3. Methodology

**Figure 1:**
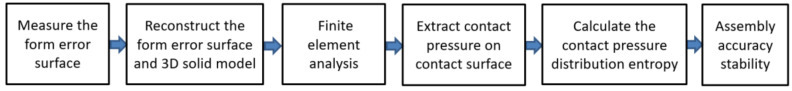
Flow chart of the improved prediction method.

Figure 1 shows the flow chart of the improved prediction method.

First, a series of measuring points characterizing the form error surface are measured using a coordinate measuring machine (CMM). The form error surface is then obtained by point cloud fitting, and fused with the 3D solid model to obtain a geometric model with form error, the modeling method of the form error model is present in [14]. Further, finite element analysis with the form error is performed, and the contact pressure of the nodes on the form error surface are extracted from the simulation results. The contact pressure distribution entropy is calculated by the method presented in subsection 2.2. The prediction results of the assembly accuracy can be obtained by comparing the contact pressure distribution entropy. The greater the contact pressure entropy and the more uniform the contact pressure distribution, the higher and more stable is the assembly accuracy.

## 3. Case Study

### 3.1. Case 1

To simplify the following discussion, the following abbreviations are used:

1. Contact pressure distribution entropy (CPE).
2. Contact strain energy distribution entropy (SEE).

**Figure 2:**
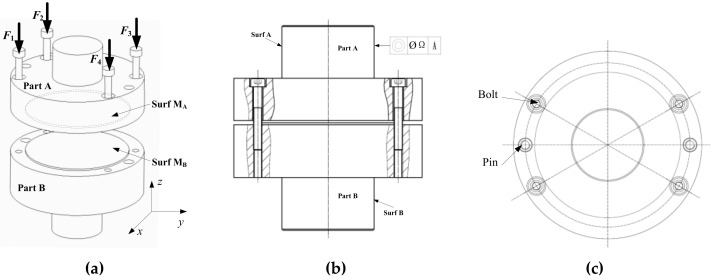
Assembly schematics of the samples. (**a**) Exploded view; (**b**) Main view; (**c**) Top view.

**Figure 3:**
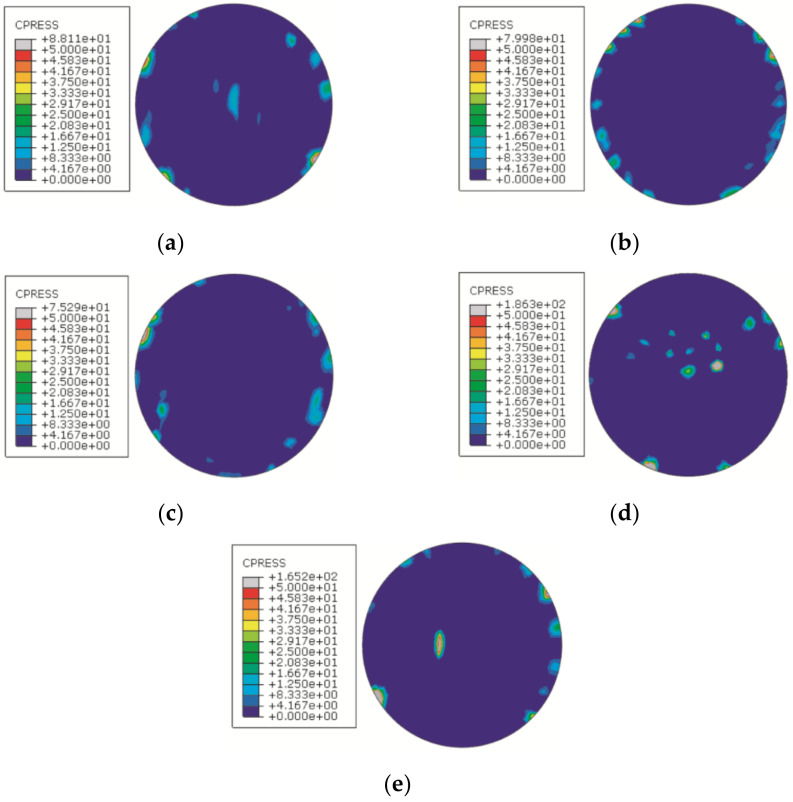
Contact pressure distribution contours of five samples. (**a**) Group 1; (**b**) Group 2; (**c**) Group 3; (**d**) Group 4; (**e**) Group 5.

Case 1 uses a sample, measurement data, and a simulation model identical to those in [11]. Figure 2 shows the composition of the sample. Parts A and B are assembled using four M4 bolts. Two pins are used for placement in the assembly process, after which they will be removed. Part A is made of carbon steel and the contact surface, *M_A_*, is obtained by grinding. Part B is made of aluminum, and the contact surface, *M_B_*, is obtained by milling. The contact surface of the sample was measured using a CMM, and a 3D model with form error was established. After assembly, the coaxiality between surfaces, *surfA* and *surfB*, was used as the verification index to characterize the assembly accuracy. Further details on this case can be obtained in [11]. Using the same simulation model that was used in the previous simulation, the contact pressure of the nodes on the contact surface was extracted, and the contact pressure distribution contour is depicted in Figure 3.

**Figure 4:**
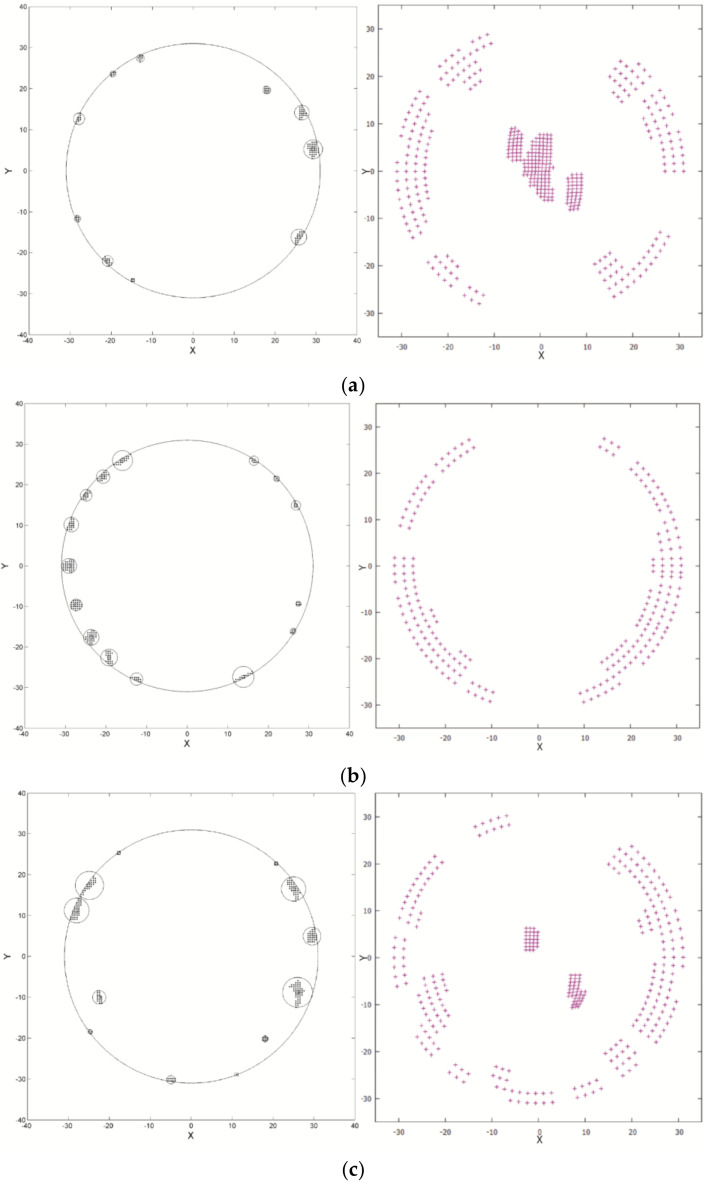

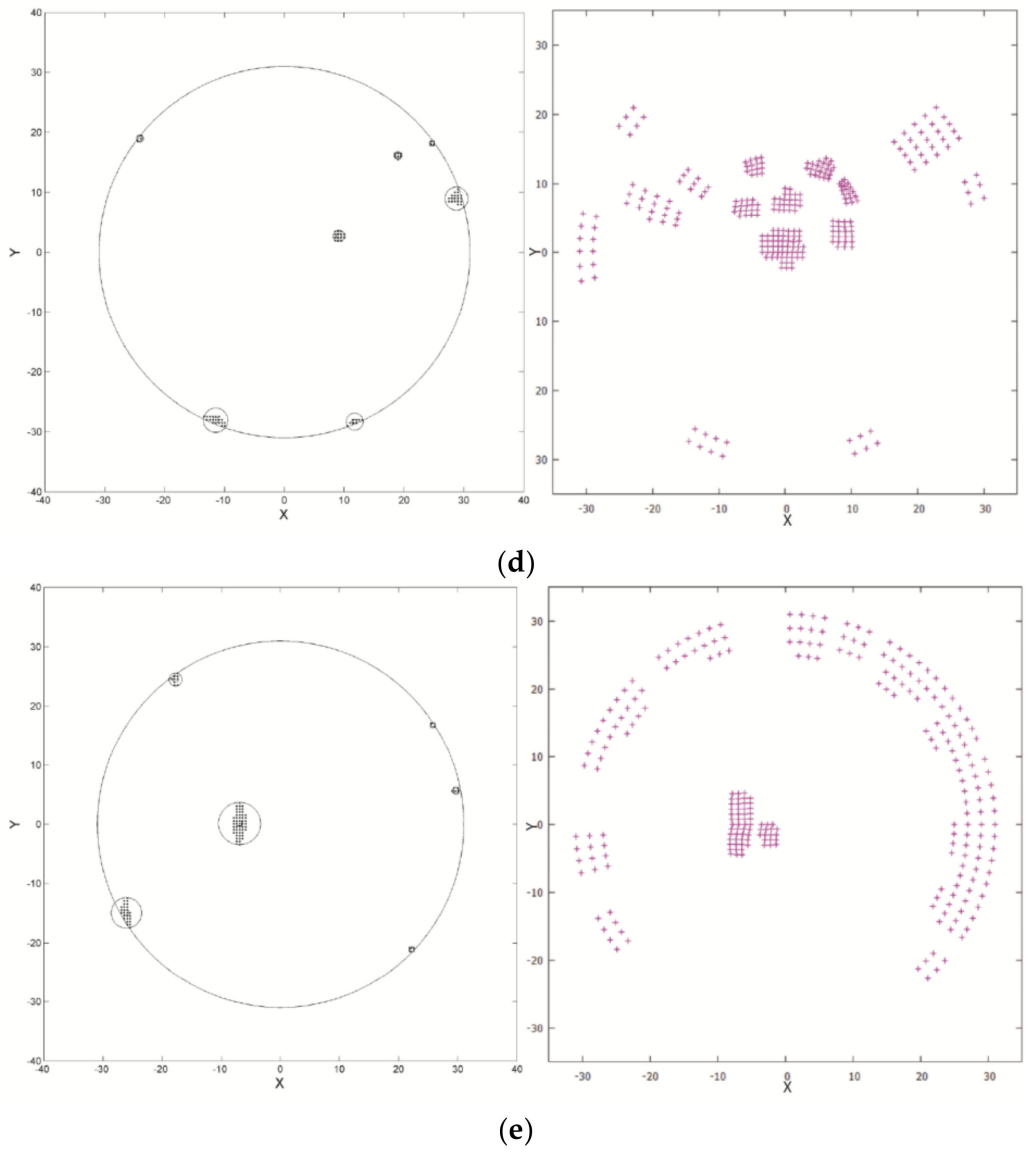
Comparison between the convex hull search results and the actual contact area. (**a**) Group 1; (**b**) Group 2; (**c**) Group 3; (**d**) Group 4; (**e**) Group 5.

Figure 4 shows the comparison between the convex hull search results presented in [11] and the contact area distribution (contact pressure greater than zero); the images on the left are the convex hull search results, and on the right is the contact area distribution, The X direction and the Y direction are identical to those shown in Figure 2a. It can be seen that the distribution of the contact pressure is similar to that of the convex hull distribution on the form error surface, indicating that the distribution of the convex hull can partly represent the contact area of the contact surface. However, in Figure 4a, 4c, and 4d, the corresponding area, where the convex hull is not distributed, indicates contact pressure distribution. This demonstrates that the convex hull search results only represent the geometric form of the sample surface, and cannot predict a deformed surface.

Table 1 shows the comparison between the original and improved methods. It can be seen that the coaxiality between *surfA* and *surfB* obtained from the experiments are consistent in the two methods. However, the evaluation process of the original method is more cumbersome than that of the proposed method.

**Figure 5:**
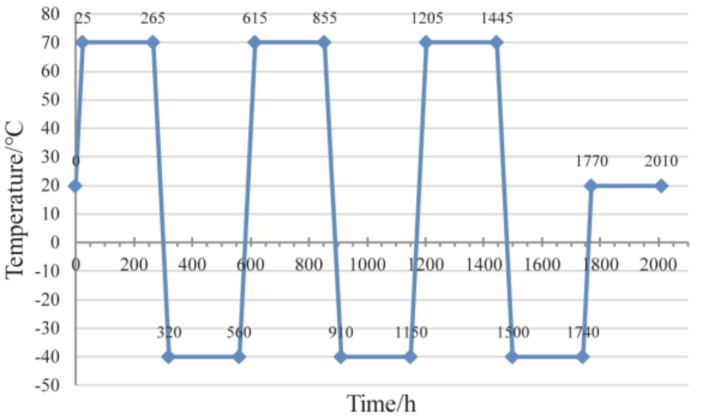
Temperature cycling curve used in experiment.

**Figure 6:**
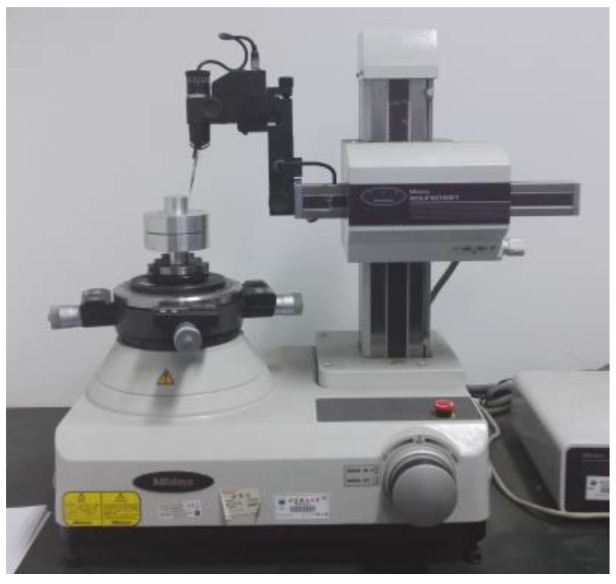
Experimental setup.

In a precision assembly process, assembly accuracy is critical. In addition, the accuracy variation of the precision parts during storage and use after assembly are important in assembly accuracy prediction. Contact pressure is the interaction force between contact surfaces, and is directly related to contact deformation and contact stress. If the contact pressure distribution is more uniform, the distribution of the contact deformation and stress of the corresponding parts should also be more uniform. Thereby, the ability of the parts to resist the influence of external loads after assembly, i.e., the ability to maintain assembly accuracy, is higher. In order to verify this, the same samples were also tested for their assembly-accuracy stability. Five groups of samples were tested in a constant-temperature test chamber after assembly, and temperature cycling was performed, as shown in Figure 5. The coaxiality between *surfA* and *surfB* of each sample before and after cyclic loading was measured and recorded by a Mitutoyo Round Test, RA-1500, as shown in Figure 6. The measurement results were used to determine whether the assembly accuracy of the sample was stable after experiencing environmental temperature changes.

Table 2 shows the contrast between the change in the coaxiality of the sample and the CPE value. It can be seen that the change trend of the assembly accuracy is consistent with that of the negative value of the CPE, i.e., the change trend of the assembly accuracy is negatively correlated with the CPE. The greater the CPE, the lesser is the variation in the assembly accuracy in the temperature cycling experiment, and the better is the stability of the assembly accuracy.

### 3.2. Case 2

**Figure 7:**
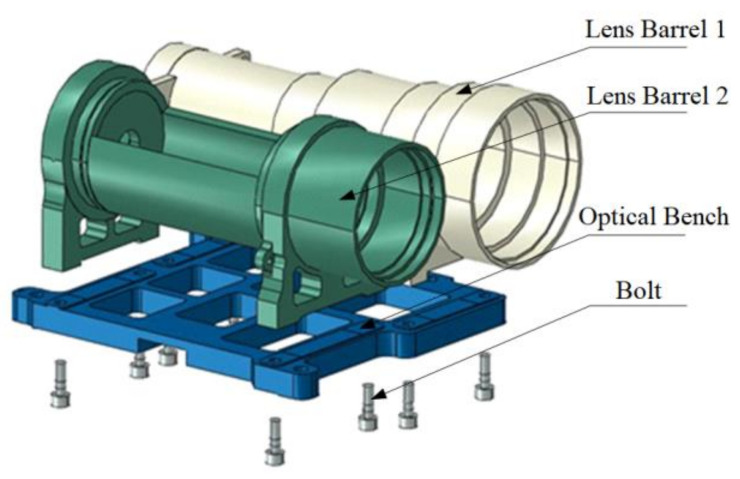
Assembly schematic of the precision optical lens.

**Figure 8:**
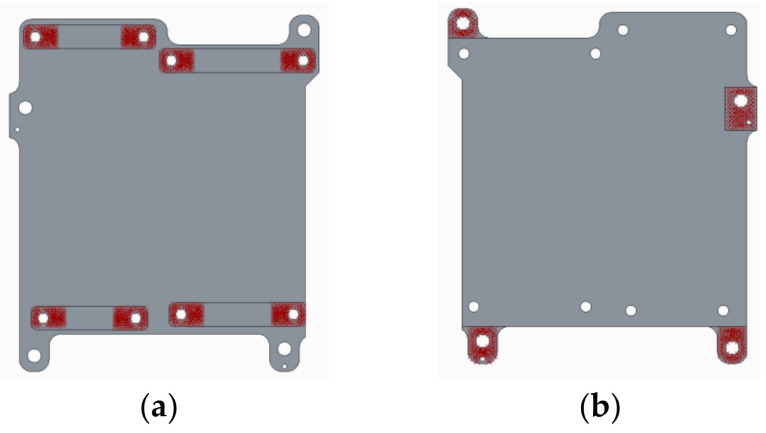
Assembly surface of optical holder: (**a**) Assembly surface with lens barrels and (**b**) Assembly surface with assembly base.

The samples used in Case 1 were for comparing the prediction effect of the two methods. However, the sample was relatively simple, and the main purpose was to verify the accuracy of the prediction method. For testing the practical application of the proposed method, a relatively complex case from an actual production process was used. The precision optical lens is a typical precision instrument, and its assembly is shown in Figure 7. The assembly consists of two lens barrels and an optical bench, connected by eight M5 bolts. The optical bench is the core part of the assembly. It is the assembly base for the two lens barrels, and also for the overall installation of the optical lens. Figure 8 shows the assembly plane of the optical holder. Figure 8a displays the assembly plane of the lens barrels and optical bench; the highlighted part is the contact part between the lens barrel and optical bench. Figure 8b displays the assembly plane between the optical bench and assembly base of the overall optical lens; the highlighted part is the contact part between the optical bench and assembly base of the overall optical lens. Therefore, the overall flatness of the four small planes in Figure 8b can affect the assembly accuracy of the precision optical lens. Due to the influence of structural deformation and processing accuracy, the assembly between the lens barrels and optical bench can inevitably lead to the warping of the optical bench assembly surface, which is related to the contact surface between the lens barrels and optical bench.

**Figure 9:**
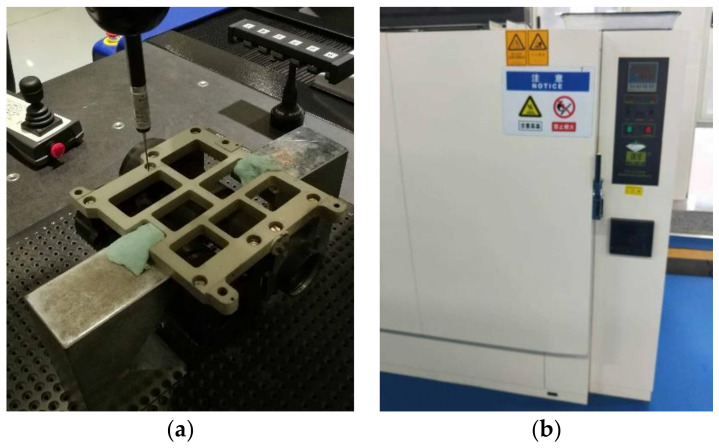
Experimental setup: (**a**) Coordinate measuring machine and (**b**) Constant-temperature test chamber.

Two optical lens samples are used in this case (Case 2). After assembly, the flatness of the optical bench-assembly surface was measured by a CMM. Temperature cycling was then conducted in a constant-temperature test chamber; the loading curve was the same as that shown in Figure 5. This temperature cycling curve was used because the operating temperature range of the optical lens was −40 to 70 °C. The flatness of the optical-bench assembly surface was again measured by the CMM, after the temperature cycling experiment. The variation in the flatness, before and after the temperature cycling experiment, was considered as the stability of the assembly accuracy. The experimental setup used in the experiment is shown in Figure 9. A 3D model with form errors on the contact surface between the optical bench and lens barrels was established, and a simulation model was established using FEM software. In order to obtain the accuracy changes caused by structural deformation alone, a model with ideal surfaces was established for comparison. The simulation parameters and boundary conditions are listed in Table 3. The contact pressure of the nodes on the contact surface were extracted from the simulation results, and the CPE was calculated using the proposed method. The calculated results are shown in Table 4.

It can be seen from Table 4 that the CPE of the ideal surface model is close to unity, indicating that the contact pressure distribution of the ideal model is highly uniform. The simulation accuracy of the ideal model changes by only 2.6 microns, which is mainly due to structural deformation. Compared to the error model, the change in the assembly accuracy of the ideal model is negligible, demonstrating that most of the accuracy change in the error model is caused by the form error on the contact surface. It can also be observed that the CPEs of the three models are negatively correlated with the variation in the assembly accuracy. These two cases prove that the proposed method is accurate and effective.

## 4. Discussion

**Figure 10:**
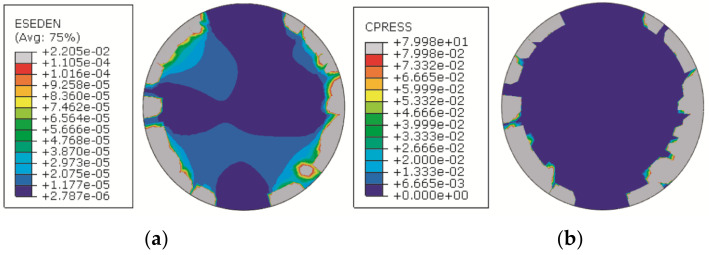
Comparison between the contact pressure and strain-energy contours. (**a**) Strain energy density contour; (**b**) contact pressure contour.

The method presented in this study is an improvement of that presented in [11]. The main difference is the change of the evaluation parameter used for calculating the entropy, from contact strain energy to contact pressure. Figure 10 shows a comparison between the contact strain energy contour and the contact pressure contour of the sample in Case 1. It can be seen that the contact pressure is zero in the center of the surface, but there is an uneven distribution of the contact strain energy in the corresponding area. This is because the strain energy represents the elastic deformation of the elastomer, and the contact pressure represents the contact deformation of the contact surface. For contact-based assemblies, contact deformation can better reflect the assembly error and the assembly error change of the assembled part than elastic deformation. Therefore, in the method presented in this paper, contact pressure is selected to predict the stability of assembly accuracy.

Information entropy, as a measure of the degree of disorder of a system, can be used to evaluate the parameter distribution uniformity. Therefore, it is used in this method to evaluate the accuracy of the precision assembly. The more uniform the contact pressure distribution is, the more uniform the contact forces and the contact deformation are. For the temperature cycling load used herein, the purpose is to simulate the accuracy retention capability of the assembly under extreme ambient temperature changes. The thermal error caused by the thermal deformation is directly related to the distribution of contact pressure, as shown in [21,22]. Under the influence of thermal expansion, the contact points on the contact surface may move relatively to each other, changing the relative positions of the assembly parts. In particular, the two examples used in this paper have different thermal expansion coefficients, which provide favorable conditions for relative motion between the contact surfaces. After the temperature cycling experiment, it was determined that for a larger CPE value, the smaller the assembly accuracy variation. This proves that the contact pressure can directly reflect the contact mechanical properties of the part; therefore, it is highly suitable to use the contact pressure as the evaluation index. In actual industrial production, the simulation of the contact mechanics can be directly performed on the part without having to perform the temperature cycling experiment, and the assembly accuracy stability of the part can be predicted.

Furthermore, the factors affecting the assembly accuracy stability include not only the ambient temperature changes, but also the changes in the assembly force with time, such as the looseness of the bolt load and the stress release of the contact surface. The effect of the relaxation of the bolt load and release of the assembly stress are manifested on the part after a long period; however, as the temperature cycling experiment in this study took a short loading time, the influence of these factors was ignored. At the same time, the two cases used in this paper are in the form of plane–plane contact. The contact surface in the form of a cylindrical surface or a conical surface may also appear in the contact surface in the actual assembly. The application of this method on these forms of surface has not been verified.

## 5. Conclusion

1. This study has presented an improved prediction method for assembly accuracy stability. By using the contact pressure as the evaluation parameter instead of the strain energy density, a simpler and more efficient prediction effect was obtained. The same simulation and experiment were performed as that in the original method, and the accuracy of the improved method was verified.
2. The contact pressure has a clearer mechanical significance than the strain energy density in the assembly process, which can be used to distinguish the actual contact area from the contact surface. Hence, the contact pressure is more suitable as the evaluation parameter than the strain energy density.
3. Through two case studies, the feasibility and effectiveness of the method were proved. Research on the impact of assembly force verification with time and application on other types of mating surfaces will be the future research direction of this method.

## Figures and Tables

**Table 1:** Comparison between the CPE value, SEE value, and the experimental results.

Group of Sample	CPE	SEE	Coaxiality between *surfA* and *surfB*/μm
1	0.84835	0.79544	102.39
2	0.84012	0.78976	141.663
3	0.82676	0.73198	233.853
4	0.80415	0.71552	240.258
5	0.77873	0.68071	402.248

**Table 2:** Comparison between the experimental result and CPE value.

Group of Sample	CPE	Coaxiality Change/μm
1	0.84835	7.86
2	0.84012	14.1
3	0.82676	21.67
4	0.80415	23.55
5	0.77873	53.26

**Table 3:** Simulation parameters and boundary conditions.

Terms	Parameters	Values
Software setting	Software	ABAQUS
	Type of contact	Surface-to-surface
	Type of simulation	Explicit
Elements	Type of element	C3D8I
	Formulation of the element	Linear
Material	Magnesium lithium alloy (lens barrels)	40 GPa (Young’s modulus)
		0.33 (Poisson’s ratio)
		2.18 × 10^−^^5^/°C
	Aluminum alloy (optical bench)	70 GPa (Young’s modulus)
		0.32 (Poisson’s ratio)
		2.38 × 10^−^^5^/°C
	Carbon steel (bolt)	210 GPa (Young’s modulus)
		0.3 (Poisson’s ratio)
		1.1 × 10^−^^5^/°C
Boundary conditions	Preload of bolts	F_1_ = F_2_ … = F_8_ = 1000 N

**Table 4:** Results of the CPE and experiments.

	Ideal Model	Error Model 1	Error Model 2
Experiment result/μm	-	18.2	14.8
Simulation result/μm	2.6	20.8	12.3
CPE	0.98876	0.92604	0.937794

## References

[1] Liu C.H. (2016). Tolerance Redistributing of the Reassembly Dimensional Chain on Measure of Uncertainty. Entropy.

[2] Davidson J.K., Mujezinovic A., Shah J.J. (2002). A new mathematical model for geometric tolerances as applied to round faces. J Mech. Des.

[3] Anwer N., Schleich B., Mathieu L., Wartzack S. (2014). From solid modelling to skin model shapes: Shifting paradigms in computer-aided tolerancing. CIRP Ann-Manuf. Technol..

[4] Schleich B., Anwer N., Mathieu L., Wartzack S. (2014). Skin model shapes: A new paradigm shift for geometric variations modelling in mechanical engineering. Comput. Aided Des..

[5] Homri L., Goka E., Levasseur G., Dantan J.Y. (2017). Tolerance analysis—Form defects modeling and simulation by modal decomposition and optimization. Comput. Aided Des..

[6] Huang W., Ceglarek D. (2002). Mode-based decomposition of part form error by discrete-cosine-transform with implementation to assembly and stamping system with compliant parts. CIRP Ann-Manuf. Technol..

[7] Guo J., Li B., Liu Z., Hong J., Wu X. (2016). Integration of geometric variation and part deformation into variation propagation of 3-D assemblies. Int. J. Prod. Res..

[8] Zhang T.Y., Zhang Z.Z., Jin X., Ye X., Zhang Z.Q. (2016). An innovative method of modeling plane geometric form errors for precision assembly. Proc. Inst. Mech. Eng. B J. Eng. Manuf..

[9] Lee N.K., Yu G., Joneja A., Ceglarek D. (2006). The modeling and analysis of a butting assembly in the presence of workpiece surface roughness and part dimensional error. Int. J. Adv. Manuf. Technol..

[10] Harsha S.P., Kankar P.K. (2004). Stability analysis of a rotor bearing system due to surface waviness and number of balls. Int. J. Mech. Sci..

[11] Fang Y., Jin X., Huang C., Zhang Z. (2017). Entropy-Based Method for Evaluating Contact Strain-Energy Distribution for Assembly Accuracy Prediction. Entropy.

[12] Babu M., Franciosa P., Ceglarek D. (2018). Shape Error Modelling and Analysis by Conditional Simulations of Gaussian Random Fields for Compliant Non-Ideal Sheet Metal Parts. Procedia CIRP.

[13] Bolotov M., Grachev I., Kudashov E. Investigation of Parts Assembly Error, Taking into Account the Deviation of the Shape of Their Surfaces. Proceedings of the International Conference on Modern Trends in Manufacturing Technologies and Equipment (ICMTMTE 2018).

[14] Zhang Z.Q., Zhang Z.J., Jin X., Zhang Q.S. (2018). A novel modelling method of geometric errors for precision assembly. Int. J. Adv. Manuf. Technol..

[15] Xiao L., Björklund S., Rosén B.G. (2007). The influence of surface roughness and the contact pressure distribution on friction in rolling/sliding contacts. Tribol. Int..

[16] Kim S.M., Lee S.K. (2001). Prediction of thermo-elastic behavior in a spindle–bearing system considering bearing surroundings. Int. J. Mach. Tool. Manuf..

[17] Germaneau A., Peyruseigt F., Mistou S., Doumalin P., Dupré J.C. (2010). 3D mechanical analysis of aeronautical plain bearings: Validation of a finite element model from measurement of displacement fields by digital volume correlation and optical scanning tomography. Opt. Lasers Eng..

[18] Zhang Y., McClain B., Fang X.D. (2000). Design of interference fits via finite element method. Int. J. Mech. Sci..

[19] Peng L., Lai X. (2010). Effect of assembly error of bipolar plate on the contact pressure distribution and stress failure of membrane electrode assembly in proton exchange membrane fuel cell. J. Power Sources.

[20] Deguchi K., Takeuchi N., Shimizu A. (2002). Evaluation of pressure uniformity using a pressure-sensitive film and calculation of wafer distortions caused by mold press in imprint lithography. Jpn. J. Appl. Phys..

[21] Yang G., Hong J., Wang N., Zhu L., Ding Y., Yang Z. Member stiffnesses and interface contact characteristics of bolted joints. Proceedings of the 2011 IEEE International Symposium on Assembly and Manufacturing (ISAM).

[22] Ito Y., Toyoda J., Nagata S. (1979). Interface pressure distribution in a bolt-flange assembly. J. Mech. Des..

[23] Nassar S.A., Barber G.C., Zuo D. (2005). Bearing friction torque in bolted joints. Tribol. T..

[24] Ramesh R., Mannan M.A., Poo A.N. (2000). Error compensation in machine tools—A review: Part II: Thermal errors. Int. J. Mach. Tool. Manu..

[25] Attia M.H., Kops L. (1980). Importance of contact pressure distribution on heat transfer in structural joints of machine tools. J. Eng. Industry.

[26] Tyfour W.R., Beynon J.H., Kapoor A. (1996). Deterioration of rolling contact fatigue life of pearlitic rail steel due to dry-wet rolling-sliding line contact. Wear.

[27] Johnson K.L. Contact Mechanics.

[28] Laskowski R., Smyk A., Rusowicz A., Grzebielec A. (2016). Determining the optimum inner diameter of condenser tubes based on thermodynamic objective functions and an economic analysis. Entropy.

[29] Mihelich M., Faranda D., Paillard D., Dubrulle B. (2017). Is Turbulence a State of Maximum Energy Dissipation?. Entropy.

[30] Muñoz-Cobo J.L., Mendizábal R., Miquel A., Berna C., Escrivá A. (2017). Use of the Principles of Maximum Entropy and Maximum Relative Entropy for the Determination of Uncertain Parameter Distributions in Engineering Applications. Entropy.

[31] Baggenstoss P. (2018). Beyond Moments: Extending the Maximum Entropy Principle to Feature Distribution Constraints. Entropy.

[32] Krantz R., Gemmetto V., Garlaschelli D. (2018). Maximum-Entropy Tools for Economic Fitness and Complexity. Entropy.

[33] Shannon C.E. (1948). A Mathematical Theory of Communication. Bell Syst. Tech. J..

[34] Melia U., Claria F., Vallverdu M., Caminal P. (2014). Measuring Instantaneous and Spectral Information Entropies by Shannon Entropy of Choi-Williams Distribution in the Context of Electroencephalography. Entropy.

[35] Xiao J., He Z.Y. (2016). A Concept Lattice for Semantic Integration of Geo-Ontologies Based on Weight of Inclusion Degree Importance and Information Entropy. Entropy.

